# Cranial ultrasonographic findings in newborns exposed to SARS-CoV-2: a single-centre cross-sectional analysis

**DOI:** 10.1186/s13052-024-01826-3

**Published:** 2024-12-05

**Authors:** Bruna Scalia, Marco Andrea Nicola Saporito, Laura Mauceri, Alessandro Valerio Saporito, Grete Francesca Privitera, Martino Ruggieri, Raffaele Falsaperla

**Affiliations:** 1 Unit of Neonatology, University Hospital Policlinico G. Rodolico– San Marco, P.O. San Marco, Viale Carlo Azeglio Ciampi, Catania, 95121 Italy; 2 Unit of Neonatology, University Hospital Policlinico G. Rodolico – San Marco, P.O. G. Rodolico, Via S. Sofia 78, Catania, 95121 Italy; 3 Department of Clinical and Experimental Medicine, Bioinformatics Unit, University of Catania, Catania, Italy; 4 Unit of Clinical Pediatrics, Department of Clinical and Experimental Medicine, University of Catania, A.O.U., P.O. G. Rodolico, Via S. Sofia 78, Catania, Italy; 5 Department of Medical Science-Pediatrics, University of Ferrara, Ferrara, Italy

**Keywords:** Neonatal SARS-CoV-2, Newborns, Cranial ultrasonography, Subependymal pseudocysts (SEPCs)

## Abstract

**Background:**

SARS-CoV-2’s potential consequences on the developing brain are still unknown. The aim of this study was to describe cranial ultrasonographic (cUS) findings in a population of newborns exposed to SARS-CoV-2 born at San Marco Hospital in Catania.

**Methods:**

Two cohort of newborns, one exposed to SARS-CoV-2 both during gestation and at birth and one unexposed, were enrolled in this cross-sectional study conducted according to the STROBE guidelines (Strenghtening the Reporting of Observational Studies in Epidemiology) and underwent cUS. We performed a statistical analysis using the Fisher’s exact test to assess whether significant differences among the two groups existed.

**Results:**

we enrolled 139 exposed newborns (62 females, 77 males with median gestational age 38.4 ± 1.9 W and median weight at birth 3142.8 ± 594.4 g) and 139 unexposed newborns (60 females, 79 males with median gestational age 38,9 ± 1.3 W and median weight at birth 3230 ± 336 g). cUS abnormalities were found in 32 exposed patients (23%) and in 23 (16.5%) unexposed patients. A statistically significant difference was found in the incidence of minor intracranial abnormalities (*p* 0.036) between exposed and unexposed patients and between newborns exposed during pregnancy and unexposed patients (*p* 0.016).

**Conclusions:**

in our experience, the incidence of minor intracranial abnormalities was higher in SARS-COV-2-exposed newborns. Our results must be taken with caution and need further confirmation in larger studies but suggest to consider performing cUS at birth in newborns exposed to SARS-CoV-2 in research contexts.

**Supplementary Information:**

The online version contains supplementary material available at 10.1186/s13052-024-01826-3.

## Introduction

Since the World Health Organization (WHO) has declared COVID-19 pandemic on March 11, 2020 [1], concerns have been raised about potential impact of SARS-CoV-2 on pregnant women and their unborn infants. The consequences of the maternal and fetal inflammatory response with the production of potentially cytotoxic cytokines, in addition to the effect of the use of antiviral medications, have not been adequately studied to date and conflicting evidences have been released on daily basis [2, 3].

Reports from large number of patients suggest that the risk of newborn infection is negligible [4–7], between 4.2% and 4.4 %; therefore, spontaneous vaginal birth, rooming-in and breast-feeding are encouraged.

On the other hand, viral infections in general during pregnancy are known to increase risk of preterm birth, spontaneous abortion, fetal growth restriction or stillbirth [8, 9].

Recent systematic reviews [10, 11] reported a higher incidence of premature birth and a higher rate of obstetrical complications in pregnancies complicated by SARS-CoV-2. Proposed mechanisms include SARS-CoV-2’s detrimental effects on the placenta, including fibrinoid deposition, enhanced inflammation and maternal vascular malperfusion that lead to fetal distress and abnormal oxygenation [12, 13]. Furthermore, not only infection but also inflammation and the subsequent cytokine storm caused by SARS-CoV-2’s infection might cause abnormal neonatal brain development which could result in neurological long-term consequences such as atypical behavioral phenotype or autistic syndromes [14, 15].

San Marco University Hospital (Catania, Sicily, Italy) has been identified as the COVID center for eastern Sicily. Therefore, all SARS-CoV-2 positive women needing obstetrical care were referred to our center. This includes expectant mothers in labor that tested positive on nasopharyngeal swabs coming from local area as well as those admitted to our Center and tested positive to nasopharyngeal routine swab test on admission, even in absence of symptoms.

The aim of the present study was to provide insight into maternal SARS-CoV-2 infections’ potential consequences on the fetus by reporting findings of cranial ultrasounds (cUS) in newborns born to mothers who had been affected by SARS-CoV-2 during gestation at our Institution and to assess whether statistically significant differences existed in the incidence of intracranial abnormalities between newborns exposed to SARS-CoV-2 and newborns born to uneventful pregnancies.

## Materials and methods

The aim of the present study was to report findings of cUS in newborns born at our Institution from mothers who had been affected by SARS-CoV-2 during gestation and to compare them with cUS findings of a similar cohort of newborns unexposed to SARS-CoV-2. For this reason, we consecutively enrolled two cohorts of newborns born at our Institution between September 2020 and September 2021, one exposed to SARS-CoV-2 and one unexposed; among the first group of patients, we later distinguished between those exposed at the time of birth (born from mothers that tested positive at delivery) and those exposed prior during pregnancy. All newborns enrolled in the study underwent cUS in the first week of life.

Inclusion criteria for “exposed patients” included:

- Newborns born to mothers that tested positive to SARS-CoV-2 infection at birth or any time during gestation;
- Newborns of any gestational age;
- Newborns who underwent nasopharyngeal swab at birth to exclude acute SARS-CoV-2 infection.

Exclusion criteria for “exposed patients” included:

- Newborns born to mothers affected by other infectious diseases during pregnancy (e.g. CMV, Rubella, Toxoplasmosis, Zika etc.);
- Newborns with prenatal diagnosis of intracranial abnormalities or genetic/syndromic diseases.

Inclusion criteria for “unexposed patients” included:

- Newborns born to mothers whose nasopharyngeal swab tested negative for SARS-CoV-2 at the time of delivery;
- Newborns born to mothers that denied a history of SARS-CoV-2 infection during gestation;
- Newborns of any gestational age;
- Regardless of the mothers’ being vaccinated for SARS-CoV-2 or not.

Exclusion criteria met those of the exposed patients.

Detailed prenatal history of each pregnant woman, including prenatal ultrasounds and TORCH analysis, was collected from medical records of mothers. To exclude other congenital infections of the fetus, data on IgG and IgM status for Toxoplasmosis, Rubella and Cytomegalovirus during pregnancy and in the last month of gestation were recorded for each expectant mother.

Of each patient enrolled in the study, the following variables were recorded: date of birth, gestational age, gestational age at the time of maternal SARS-CoV-2 infection, maternal symptoms related to SARS-CoV-2, cUS findings, birth weight, neonatal complications, maternal and obstetrical complications during pregnancy, antepartum or postpartum (e.g. maternal diabetes, hypertension, medications use during pregnancy, substance abuse during pregnancy, neonatal jaundice, maternal autoimmune diseases).

Once born, according to an internal protocol, newborns born from mothers that tested positive at the time of delivery, underwent biofire film-array on nasopharyngeal swab at birth to exclude SARS-CoV-2 infection and simultaneously detect- and, hence, exclude- other viral DNA/RNAs and was consecutively enrolled for cUS. Newborns born to mothers that tested negative for SARS-CoV-2 weren’t tested with nasopharyngeal swab.

Also, all infants exposed to SARS-CoV-2 underwent newborn neurological examination (NNE) as a part of routine care and were enrolled in a neurological follow-up program which is currently undergoing, regardless of the cUS results.

All cUS images of newborns enrolled were taken in standard coronal and sagittal planes through the anterior fontanel using the same portable ultrasound system with a 6–10 MHz transducer (Vivid S5, GE Medical Systems, Solingen, Germany) by three neonatologists with more than ten years-experience in cranial ultrasonography.

cUS findings were categorized into three groups according to classifications used in similar studies [16, 17]:

- Normal or nonsignificant (NS) findings: mild ventricular asymmetry, mild periventricular echogenicity, mild frontal or occipital horn prominence, choroid plexus irregularity, septum pellucidum cysts.
- Minor abnormalities: thalamic-striatal vessels’ echogenicity, enlarged cysterna magna, choroid plexus or subependymal cysts (SEPCs), mild ventricular enlargement, choroid plexus hemorrhage, ventricular irregularity, periventricular echogenicity and subependymal echogenicity, frontal horn cysts.
- Major abnormalities: anomalies of the corpus callosum, ventriculomegaly, hydrocephalus, porencephalic cyst, arachnoid cysts, grade III intraventricular hemorrhage or any grade IVH plus periventricular venous infarct, subdural hemorrhage, cerebellar hemorrhages.

The study conformed to the Strenghtening the Reporting of Observational Studies in Epidemiology (STROBE) Statement and all points of the checklist have been respected.

## Statistical analysis

Quantitative data (e.g. newborns gestational age, weight at birth, Apgar scores) were expressed as means and standard deviations; qualitative data (e.g. gender, mode of delivery, cranial ultrasonographic finding) were expressed as percentages.

Categorical variables were compared by using the Chi-Squared or Fisher exact test, as appropriate. Statistical analysis was performed using IBM SPSS 23.0 and GraphPad Prism 9 software. A *P*-value of less than 0.05 was considered significant.

## Results

In the present study, ultrasonographic data of 278 newborns were collected at San Marco University Hospital between September 2020 and September 2021; in detail, cranial ultrasonographic findings of 139 SARS-CoV-2-exposed patients and of 139 unexposed patients were reported.

Demographic features of the population included are listed in Tables 1 and 2, that also underlines the similarities between the patients included in the two groups except for exposure to SARS-CoV-2.

**Table 1:** Demographic features of the population included

Gender	Total population of newborns exposed to SARS-CoV-2 N. of patients (*N*, %)	SARS-CoV-2 at birth (*N*, %)	SARS-CoV-2 during pregnancy (*N*, %)	Newborns non exposed to SARS-CoV-2 (*N*, %)
*Female* *Male*	62 (44,6%) 77 (55,4%)	46 (40,7%) 67 (59,3%)	15 (57,7%) 11 (42,3%)	60 (43,1%) 79 (56,9%)
**Gestational age at birth (weeks)**				
* M* ± *SD*	38.4 ± 1.9	38.3 ± 2.02	38.7 ± 1.3	38,9 ± 1.3
* Mdn*	39	39	39.05	39.2
* Mo*	39	39	38.3	38.6
**Weight at birth (grams)**				
* M* ± *SD*	3142,8 ± 549,4	3129,5 ± 585,6	3200,7 ± 354, 4	3230 ± 336
* Mdn*	3200	3200	3187,5	3210
* Mo*	3400	3400	2940	3010
**Prematurity rate**				
Total population	20 (14,3%)	16 (80%)	4 (20%)	18 (12,9%)
GA 34 – 37 W		14	4	
GA 29–33 W		2		

*Mdn* median, *M* mean, *Mo* mode, *SD* standard deviation, *GA* gestational age, *N* number, *W* weeks

**Table 2:** Demographic features of the population of preterms included

**Gender**	**Total population of newborns exposed to SARS-CoV-2** **N. of patients (*N*)**	**SARS-CoV-2 at birth** **(*N*)**	**SARS-CoV-2 during pregnancy (*N*)**	**Newborns non exposed to** **SARS-CoV-2** **(*N*)**
*Female*	12	9	3	9
*Male*	8	7	1	9
**Gestational age at birth (weeks)**				
* M* ± *SD*	35.2 ± 1.8	34.9 ± 2.1	35.3 ± 1	34.1 ± 2.6
* Mdn*	35.8	35	35.6	35.2
* Mo*	36	35	35.5	35
**Weight at birth (grams)**				
* M* ± *SD*	2800 ± 353,2	2630 ± 536,3	2730 ± 294	2680 ± 492,5
* Mdn*	2650	2580	2790	2700
* Mo*	2700	2600	2750	2650

*Mdn* median, *M* mean, *Mo* mode, *SD* standard deviation, *GA* gestational age, *N* number, *W* weeks

**Fig. 1:**
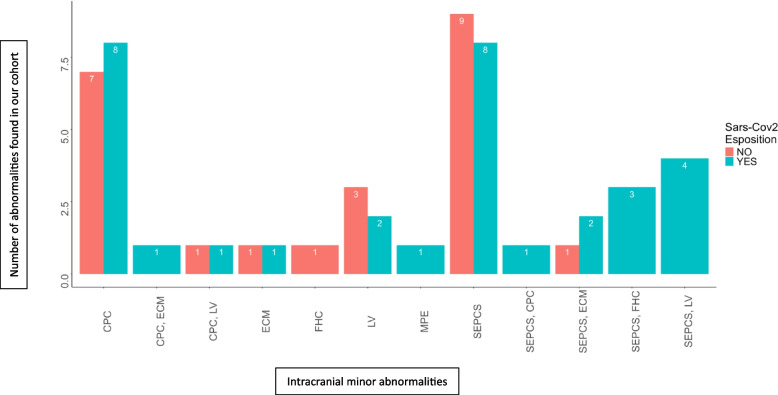
Number and type of minor intracranial abnormalities in our cohort of exposed and unexposed patients

Ultrasonographic findings of each group are listed in Tables 3 and 4, respectively and displayed graphically in Fig. 1. In summary, among patients exposed to SARS-CoV-2, we found 32 neonates (23%) with cUS abnormalities (10 in the prenatally exposed and 22 in the perinatally exposed group) and 107 neonates (77%) with normal cUS (16 in the prenatally exposed and 22 in the prenatally exposed group); 44/46 (95.6%) of the abnormalities found were minor. In detail, we identified 11 subependymal cysts (SEPCs), 11 choroid plexus cysts (CPC), 7 lenticulostriate vasculopathy (LV), 3 frontal horn cysts (FHCs), 2 enlarged cisterna magna (ECM), 2 mild periventricular echogenicity (MPE), and 1 case of corpus callosum abnormality.

**Table 3:** cUS findings among 113 patients exposed to SARS-CoV-2

At birth			During Pregnancy	
	N. of patients	% of patients	N. of patients	% of patients
**Number of patients with abnormalities**	**22**	**19.5%**	**10**	**38.4%**
**Total number of abnormalities**	**32**		**14**	
**Number of normal cUS**	**91**	**80.5%**	**16**	**61.6%**
**Minor abnormalities**				
• Subependymal cysts	10	8.8%	8	30.7%
• Left	6		2	
- Right	4		2	
- Multicystic	1		4	
- Bilateral	/		4	
Choroid plexus cysts	10	8.8%	1	3.8%
- Single	8		1	
- Multiple	1			
- Bilateral and multiple	1			
Frontal horn cysts	/		3	11.5%
Lenticulostriate vasculopathy	5	4.4%	2	7.6%
Enlarged cisterna magna	2	1.7%	/	
Mild ventricular dilatation	/		/	
Mild periventricular echogenity	2	1.7%	/	
Ventricular asymmetry	/		/	
Widening of the interhemispheric scissure	/		/	
Corpus callosum abnormalities	1	0.9%	/	
**Major abnormalities**				
Arachnoid cyst	1	0.9%		
Cerebellar hemorrhage	1	0.9%

**Table 4:** cUS findings in 139 newborn patients non-exposed to SARS-CoV-2 during pregnancy

N. of patients		% of patients
**Number of patients with abnormalities**	**23**	**16,5%**
**Total number of abnormalities**	**23**	**83,5%**
**Number of normal cUS**	**116**	
**Minor abnormalities**		
• Subependymal cysts	10	43,7%
• Choroid plexus cysts	8	5,75%
Frontal horn cysts	1	0,71%
Lenticulostriate vasculopathy	3	2,1%
Enlarged cisterna magna	1	0,71%
Mild ventricular dilatation	/	
Mild periventricular echogenity	/	
Ventricular asymmetry	/	
Widening of the interhemispheric scissure	/	
Corpus callosum abnormalities	/	
**Major abnormalities**	/

Of the 113 newborns born to mothers SARS-CoV-2 positive at the time of delivery, 22 (19.5%) had cUS abnormalities, 20 of which (90.9%) were minor (Table 3). One patient with thickened corpus callosum was a term female newborn later identified as Crouzon Syndrome, a condition that can’t be indeed related to SARS-CoV-2’s infection. Two patients had major abnormalities including arachnoid cyst (0.8%) in a patient with dysmorphic features and cerebellar hemorrhage (0.8%) in a preterm with severe respiratory distress syndrome and early onset sepsis (EOS).

Of the 26 newborns born to mothers that tested positive to SARS-CoV-2 during pregnancy, 10 had minor abnormalities on cUS, none had major abnormalities. Half of the patients included were exposed during the second trimester, between 18 and 24 weeks of pregnancy: 8 patients were exposed during the first trimester and 5 patients were exposed between 25 and 36 W. All the 10 patients with minor abnormalities on cUS (details in Table 3) were exposed to SARS-CoV-2 during the second trimester.

Risk factors and comorbidities of newborns with minor cUS abnormalities included small for gestational age (SGA) in 8 (25%), mild to moderate prematurity in 8 (25 %), maternal symptoms in 4 (12.5%).

**Fig. 2:**
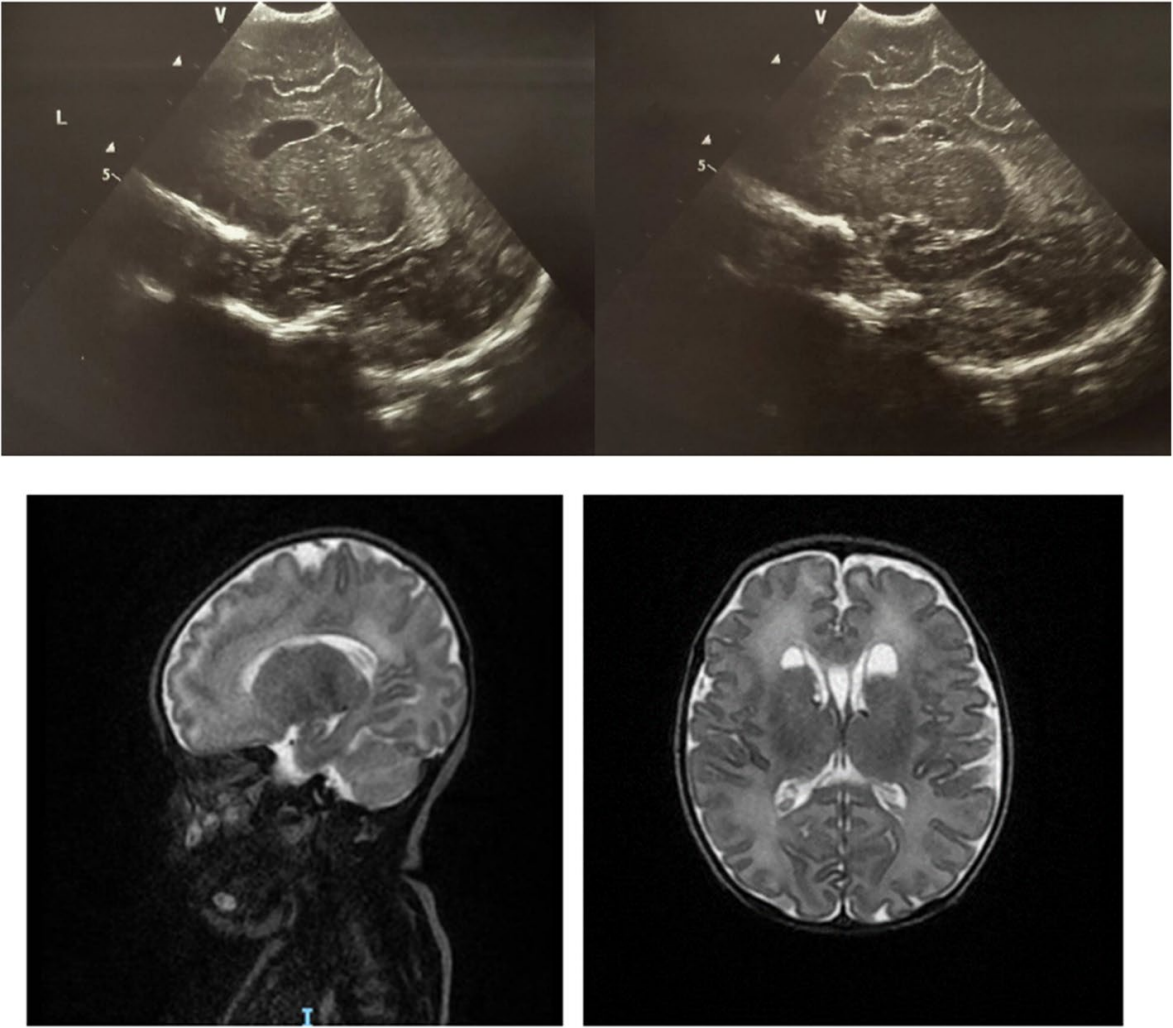
cUS and T_2_-weighted sequences of the brain MRI of a term female newborn exposed to SARS-CoV-2 in utero at 22 W showing bilateral FHCs and bilateral subependymal cysts

**Fig. 3:**
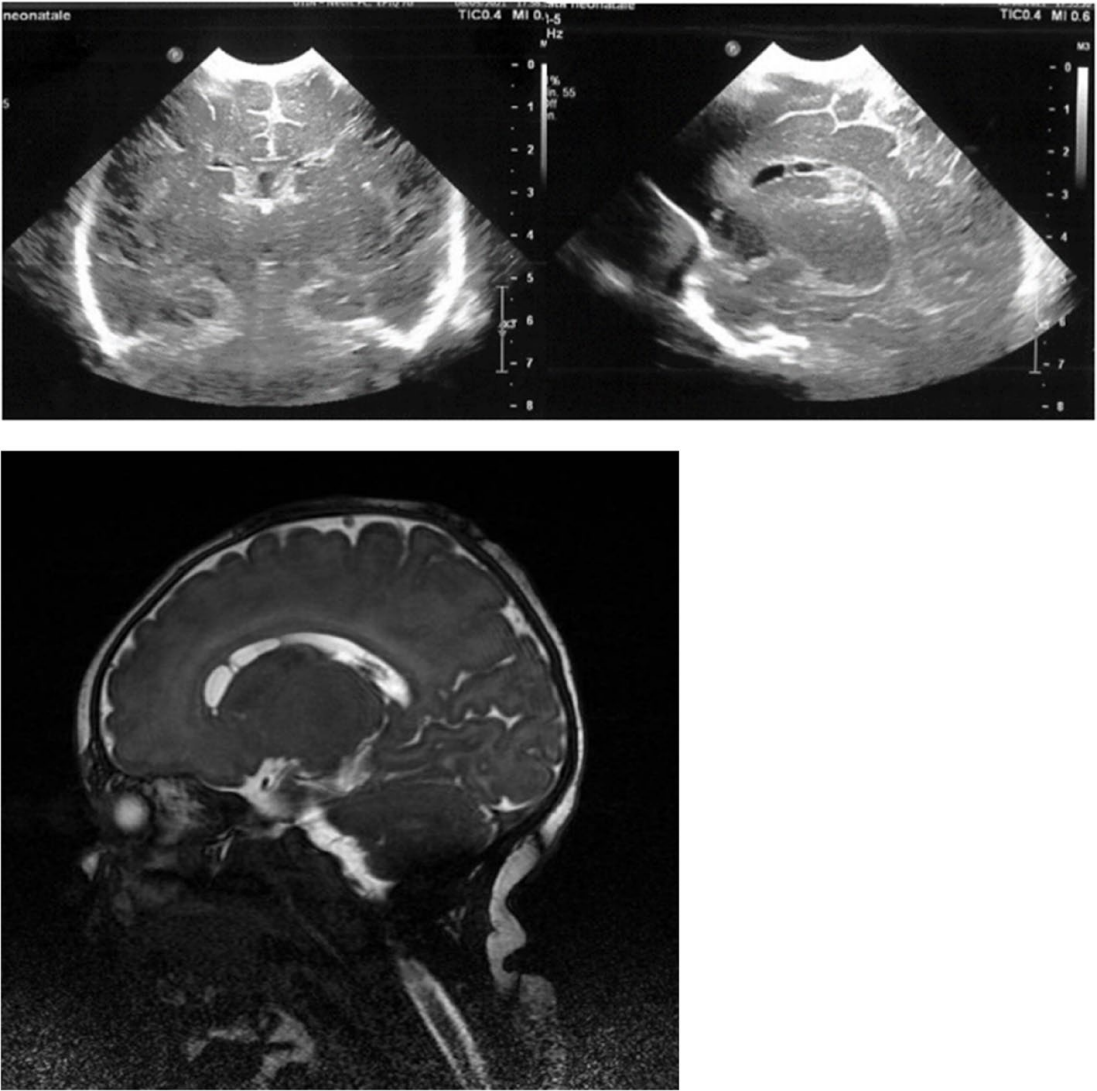
cUS and brain MRI of a term female newborn exposed to SARS-CoV-2 in utero at 22 W showing bilateral FHCs and bilateral subependymal cysts involving the caudate nucleus

**Fig. 4:**
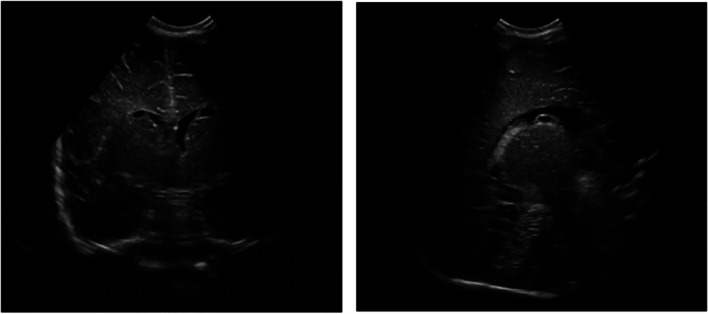
cUS of a term male newborn exposed to SARS-CoV-2 in utero at 19 W showing bilateral FHCs and left subependymal cysts

Interestingly, in a subgroup of newborns whose mothers’ tested positive for SARS-CoV-2 in the second trimester (22, 22 and 19 weeks, respectively), we identified three patients with FHCs and simultaneous multiple, large, bilateral SEPCs. TORCH screening was inconclusive and pregnancy was uncomplicated by other events. Patients, whose details are summarized in Figs. 2, 3 and 4, respectively, are currently undergoing timely neurological evaluations and, to date, have shown no significant deviation from normality.

Among the cohort of unexposed patients, we found 23 neonates (16,5%) with cUS abnormalities, all of which were minor; in detail, we identified 10 SEPCs, 8 CPC, 3 LV, 1 FHC and 1 ECM (Table 4).

**Fig. 5:**
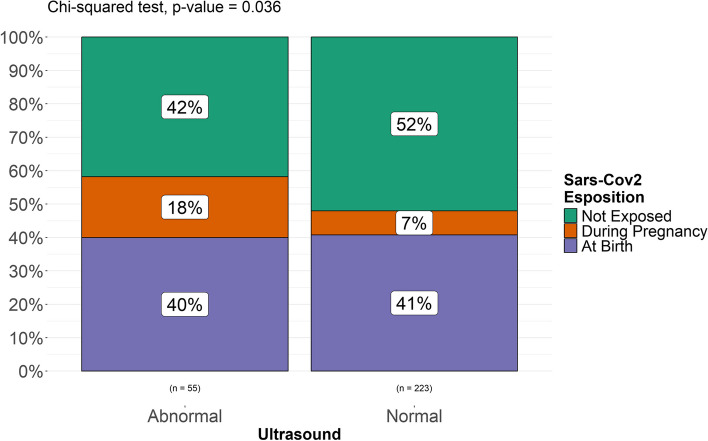
Differences among patients exposed and unexposed to SARS-CoV-2

**Fig. 6:**
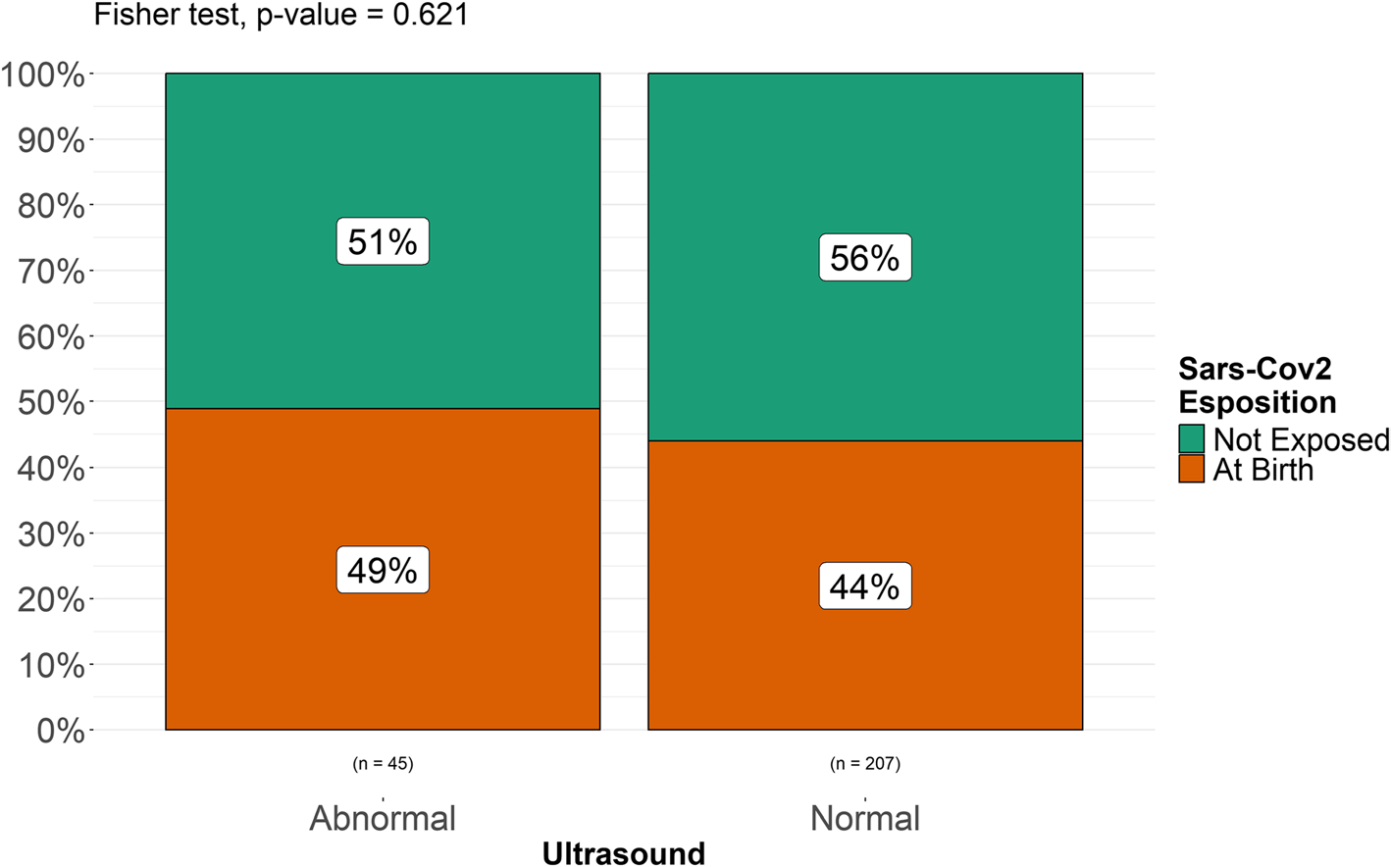
Differences between patients exposed at birth and not exposed

**Fig. 7:**
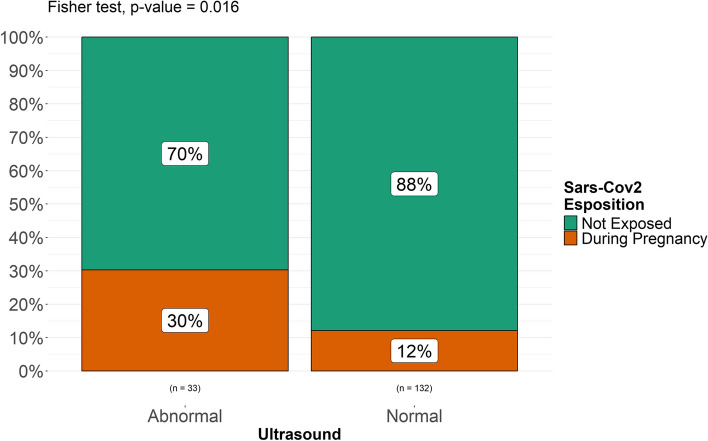
Differences between newborns exposed during pregnancy and unexposed newborns

**Fig. 8:**
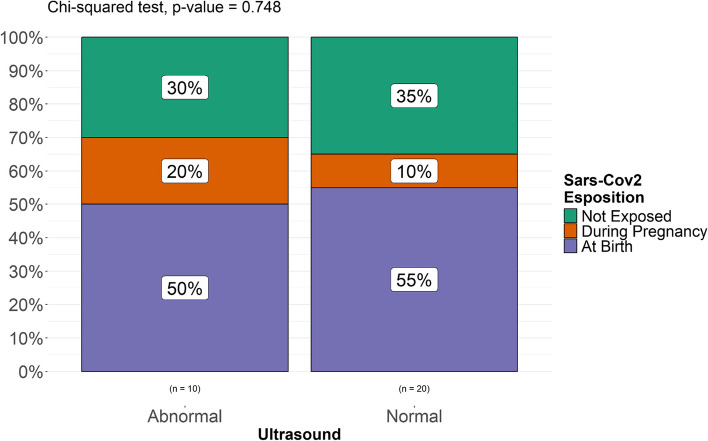
Differences between preterm exposed to SARS-CoV-2 (at birth or in utero) and unexposed preterm

A Fisher’s exact test was performed to compare two categorical variables (“exposed” vs “non exposed”, “exposed prenatally” and “exposed at birth”, “exposed at term” vs “exposed preterm”, “exposed preterm” vs “non exposed” and “non exposed preterm”) and assess the possible association between exposure to SARS-CoV-2 and intracranial minor abnormalities in each group of patients included and found a statistically significant difference in the incidence of minor intracranial abnormalities (*p. *value 0.036) between exposed (both prenatally and at birth) and unexposed patients (Fig. 5). No statistically significant difference was found between newborns exposed at birth and unexposed patients (*p. *value 0.62, Fig. 6). A statistically significant difference (*p. *value 0.016) was found between patients exposed during pregnancy and unexposed patients (Fig. 7). It must be considered, though, that the numerosity of the sample is highly different and this might represent a bias in analyzing differences. When stratified for gestational age, no statistically significant correlation was found between prematurity and a higher rate of intracranial abnormalities in our populations of both exposed and unexposed patients (Fig. 8).

## Discussion

SARS-CoV-2 infection in pregnant women has long been believed to be harmless for fetuses. However, increasing evidence supports the hypothesis that a systemic inflammatory response to the SARS-CoV-2 virus and its consequent endothelial damage might be implicated in leading to placental malperfusion, described in up to 46 % of cases of pregnant women affected by SARS-CoV-2 and to subsequent fetal distress [12, 14, 18, 19]. Recent studies have also underlined the potential detrimental role of inflammation, even in absence of infection [20, 21] suggesting that fetal distress might be the consequence of the massive cytokine storm headed by interleukin-6 (IL-6) typical of SARS-CoV-2’s infection and even more pronounced in pregnant ladies [22]. Cytokines may enter fetal brain by crossing blood-brain barrier or by altering its permeability and negatively impact on synaptogenesis [23, 24]. This hypothesis is reinforced by older evidences that supported a relationship between maternal elevated IL-6 and decreased cognition at 12 months [25], introducing the idea of a possible correlation between maternal inflammation and both structural and functional disorders of the brain leading to neuropsychologic disorders of the offspring [13, 15, 25, 26]. However, it still is unclear whether and to what extent fetal and neonatal blood-brain barrier functions as a protective factor in blocking inflammatory cytokines. Unfortunately, current evidences are still based mainly on case reports [27–31]. Our data aimed to add knowledge to SARS-CoV-2’s potential detrimental effects on the fetus by reporting experiences of cUS in newborns exposed to SARS-CoV-2 and by confronting their findings with those of an unexposed cohort of similar newborns. Among exposed newborns we identified brain abnormalities on cUS in 32 newborns (23%), while 107 patients (77%) had a normal cUS or the abnormalities were considered insignificant. 44/46 (95.6%) abnormalities found were considered to be minor according to classifications used in large cohort-based cUS findings studies among healthy term newborns. Interestingly, 53.8 % of cUS abnormalities were found in newborns exposed to SARS-CoV-2 during pregnancy, whereas only 28.3 % of the abnormalities were found in newborns exposed to SARS-CoV-2 at the time of birth. Furthermore, all the patients with prenatal exposure to SARS-CoV-2 and cUS abnormalities were exposed during the second trimester, suggesting this time might be a vulnerable period for the developing brain. Among unexposed patients, instead, we found 23 neonates (16,5%) with cUS abnormalities, all of which were minor. A statistical analysis to detect any possible correlation between SARS-CoV-2 and minor abnormalities in each group of patients and in subgroups of them (e.g. preterms), found that a statistically significant correlation (*p* 0.036) existed between overall exposed patients (both prenatally and at birth, at term and preterm altogether) and unexposed patients, suggesting exposure to SARS-CoV-2 might relate with a higher incidence of intracranial abnormalities. Furthermore, a statistically significant difference (*p 0.016*) was found between the incidence of cUS abnormalities in newborns exposed during pregnancy vs newborns exposed at birth suggesting that prenatal exposure might relate with a higher risk of developing minor intracranial abnormalities. On the other hand, no statistically significant difference was found when comparing other subgroups of patients (e.g. preterms exposed vs term exposed newborns, preterms exposed vs term exposed newborns, exposed at birth vs unexposed).

When comparing findings of our population with those of the general healthy population available in literature, we found that the most common minor ultrasonographic findings abnormalities were similar in terms of incidence [16, 17, 32] except for newborns prenatally exposed to SARS-CoV-2 during pregnancy who had a higher incidence of minor cUS abnormalities (38.4%) compared both to newborns exposed to SARS-CoV-2 at birth and to unexposed newborns, both in our experience and in the literature (Table 5) [16, 17]. The incidence of SEPCs in this subgroup of patients is particularly high; [16, 17, 32] interestingly, we found three cases of newborns born to mothers that tested positive for SARS-CoV-2 between the 19^th^ and the 22^nd^ W of gestation who presented with FCHs and bilateral, multicystic subependymal cysts. The incidence of FHCs associated to SEPCs in our experience is 2.15%. No reports on potential causes or consequences of this association are available in the literature. SEPCs’ pathogenesis remains mostly unclear, although several antenatal risk factors including infections in general have been identified as responsible in some cases [33]. Also their nature, historically believed to be “benign” has recently been discussed, suggesting that neonatal SEPCs might relate to a higher rate of neuropsychiatric disorders later in life [34–36]. Changes in placental blood flow induced by SARS-CoV-2 might lead to a dysregulation of the highly vascularized germinal matrix, resulting in its infarction and subsequent SEPCs formation [37].

Our results -in line with few previous reports from the literature- reinforce the suspicion that SARS-CoV-2 infection during pregnancy might be associated with encephalic changes and in particular with a higher incidence, compared to unexposed newborns, of minor abnormalities and hence, open up a spectrum of research possibilities regarding SARS-CoV-2’s effects on fetal, neonatal and childhood health; indeed, it must be said that minor intracranial abnormalities do not have a clear and linear association to neurological outcomes and in clinical practice parents must be advised, in order to avoid raising anxiety and concerns, that minor abnormalities do not define a neurological abnormality or a future deviation in neurodevelopmental milestones acquisition; also, we acknowledge that our results must be interpreted with caution both because of the small number of patients included in the project and because of its several limitations (See: Strengths and limitations). Nevertheless, we believe our findings suggest the need for larger prospective studies to be performed to determine SARS-CoV-2 potential detrimental effect on the developing brain and, in the meantime, justify the choice to perform such a non-invasive and minimally expensive examination as cUS at birth and, when possible, to enroll newborns exposed to SARS-CoV-2 in long-term neurodevelopmental follow-up programs.

**Table 5:** Incidence rate per type of cUS anomalies in our population and in general population reported in literature

	**Our population of exposed patients**	**Our population of unexposed patients**	**Hsu et al., 2012**	**Lin et al.,** **2020**
Number of patients included	139	139	3186	11,681
Incidence of cUS minor abnormalities	23%	16.5%	6.3%	17.3%
SEPCs	12.9%	7.1%	3.1%	8.99%
CPCs	7.9%	5.7%	0.38%	2.43%
FHCs	2.1%	0.7%	N/A	1.8%
LV	5%	2.1%	N/A	2.34%
ECM	1.4%	0.7%	0.5%	1.04%
Increased echogenicity	1.4%	0%	0.28%	N/A
Incidence of cUS major abnormalities	1.4%	0%	0.06%	0.28%

## Strengths and limitations

The current study has several strengths. This is, to our knowledge, the first report of cUS findings in newborns exposed to SARS-CoV-2. All patients included underwent nasopharyngeal swab testing for SARS-CoV-2 at birth to exclude active infection and other viral infections. A strict enrollment protocol was adopted for this study and timing and methodology of the cUS was homogeneous for all patients. Each of the pregnant ladies included had been tested extensively for TORCH, hence the possibility of other infections misleading cUS is negligible. The comparison with a similar group of unexposed patients gives strength to the paper but it must be said that the possibility to assess the influence of other risk factors (e.g. gestational age) on the incidence of minor intracranial abnormalities is limited and the two groups, though homogeneous in numbers and features, might not be representative of a larger population. Also, the study has other limitations including the different numerosity of cohorts included, the small number of patients enrolled in the prenatally exposed group and the fact that unexposed patients were defined such based on the anamnesis and the negativity of the nasopharyngeal swab at birth and not on the mothers’ serologic status evaluation. Also, histopatological examination of the placentas were not included as a result in the present paper but their investigation might have added some interesting data and strength to our results.

## Conclusions

In our population of newborns exposed to SARS-CoV-2 compared to a population of unexposed newborns, the incidence of intracranial abnormalities on cUS was higher than that of the control group in a statistically significant way and higher than that of the general population as described in large cohort studies. In newborns exposed to SARS-CoV-2 during the first two trimester of pregnancy, the incidence of minor ultrasonographic abnormalities was even higher (38.4%) and three previously unreported cases of association between FHCs and SEPCs have been described, pointing to a possible association between these cUS findings and mid-gestation SARS-CoV-2’s exposure. Our results, together with evidence from the literature of placental histopathological findings in expectant mothers infected with SARS-CoV-2 during pregnancy, suggest a possible connection between SARS-CoV-2 infection and a higher rate of minor cUS abnormalities in newborns, which needs further confirmation in larger prospective studies or randomized controlled trials; however, considering that cUS is a non-expensive, non-invasive bedside diagnostic tool, we suggest performing cranial ultrasound and periodic neurological follow-up evaluations to patients exposed to SARS-CoV-2 in utero or at birth could be useful in further clinical studies and should be proposed to parents in a research context.

## Supplementary Information

Supplementary Material 1.

## Data Availability

The datasets used and/or analysed during the current study are available from the corresponding author on reasonable request.
